# INEQUALITIES IN OLD AGE WELL-BEING: THE ROLE OF REGIONAL DEPOPULATION AND SOCIAL AND MATERIAL CONDITIONS

**DOI:** 10.1093/geroni/igad104.0223

**Published:** 2023-12-21

**Authors:** Martina Brandt, Alina Schmitz

**Affiliations:** TU Dortmund University, Dortmund, Nordrhein-Westfalen, Germany; TU-Dortmund, Dortmund, Nordrhein-Westfalen, Germany

## Abstract

Material conditions and social integration are important predictors of wellbeing in old age. It can be expected that older individuals who reside in depopulating areas experience declines in well-being, as their property loses value, their social networks are shrinking, and public infrastructure is lacking. We conduct comparative analyses of Germany and Poland, two countries that show significant differences with regard to the economic and social situation of the older population. We expect that regional depopulation is more detrimental to well-being in Poland, as older individuals rely more strongly on informal support and individual resources in old age. We apply multilevel regression models to investigate the interrelations between overall life satisfaction, social integration, material conditions and regional factors. We combine micro-data from the Survey of Health, Ageing and Retirement in Europe (SHARE) with macro-data on regional characteristics (depopulation, public infrastructure and economic development). Our preliminary analyses suggest that a substantial part of inequalities in old age well-being can be traced back to regional differences in the level of depopulation. Social integration and material conditions are important mediators of this association. Additionally, regional differences in care and health infrastructure are important predictors of well-being – especially for older adults in need of financial and social support. Regional depopulation can be detrimental to well-being in later life due to material hardship, low social integration and lacking public infrastructure. In the next steps, we will investigate whether there are differences at the country level by comparing Germany and Poland.

